# Liraglutide treatment is associated with progression of coronary artery fibrous plaque: a prospective 1-year follow-up study in asymptomatic patients with type 2 diabetes

**DOI:** 10.1186/s12872-023-03228-5

**Published:** 2023-04-28

**Authors:** Laurits Juhl Heinsen, Gokulan Pararajasingam, Thomas Rueskov Andersen, Søren Auscher, Hussam Mahmoud Sheta, Helle Precht, Kalle Brunebjerg Engdam, Jørgen Hangaard, Jess Lambrechtsen, Filip Krag Knop, Kenneth Egstrup

**Affiliations:** 1 Cardiovascular Research Unit, Odense University Hospital Svendborg, Baagøes Allé 15, Svendborg, 5700 Denmark; 2 Department of Cardiology, Odense University Hospital Svendborg, Baagøes Allé 15, Svendborg, 5700 Denmark; 3 Department of Endocrinology, Odense University Hospital Svendborg, Baagøes Allé 15, Svendborg, 5700 Denmark; 4 Center for Clinical Metabolic Research, Gentofte Hospital, University of Copenhagen, Gentofte Hospitalsvej 7, 3rd floor, Gentofte, 2820 Denmark; 5 Steno Diabetes Center Copenhagen, Gentofte, Denmark; 6 Department of Clinical Medicine, Faculty of Health and Medical Sciences, University of Copenhagen, Copenhagen, Denmark; 7 Institute of Regional Research, University of Southern Denmark, Winsløwsparken 19, 5000 Odense C, Denmark; 8 Department of Radiology, Lillebaelt Hospital, University Hospitals of Southern Denmark, Sygehusvej 24, 6000 Kolding, Kolding, Denmark

**Keywords:** Type 2 diabetes mellitus, Coronary atherosclerosis, Glucagon-like peptide 1 receptor agonist, Liraglutide, Coronary computed tomography angiography

## Abstract

**Objective:**

The objective of this study was to assess the association between clinically indicated liraglutide treatment and coronary artery plaque progression during 1-year follow-up in asymptomatic diabetes.

**Methods:**

Patients were divided into a group receiving liraglutide (Lira+) and a group not receiving liraglutide (Lira-). Coronary computed tomography angiography (CCTA) was performed to assess total atheroma volume (TAV) and subtypes of plaque volumes (dense calcium, fibrous, fibrous-fatty, and necrotic core plaque) and the plaque progression during one year follow-up.

**Results:**

Fifty-five patients (27%) receiving liraglutide and 149 (73%) how did not were included. Changes in TAV during 1-year of follow-up were similar in the two groups (38 ± 180 (Lira+) vs. -1 ± 160 mm^3^ (Lira-), *P* = 0.13). A greater increase in fibrous plaque volume was seen in the Lira + vs. the Lira- group (34 ± 129 vs. -2 ± 101 mm^3^, *P* = 0.04). Changes over 1-year in the other plaque subtypes were similar in the two groups. Treatment duration of liraglutide was not associated with changes in TAV.

**Conclusion:**

In patients with T2D without known prior coronary artery disease, liraglutide treatment was associated with a significant increase in coronary artery fibrous plaque volume during 1-year follow-up.

## Introduction

Cardiovascular disease (CVD) is the main cause of morbidity and mortality in type 2 diabetes (T2D) and patients with T2D have twice the risk of dying from CVD compared to individuals without diabetes [1]. Focus on traditional risk-factor modulation has proven efficient [2], but a further reduction in CVD is still needed. The cardiovascular outcome trial LEADER showed that the occurrence of major adverse cardiovascular events (defined as nonfatal myocardial infarction, nonfatal stroke, and death from CVD) in high-risk patients with T2D was reduced by treatment with the glucagon-like peptide 1 (GLP-1) receptor agonist liraglutide when compared to placebo added to standard of care [3]. The mechanisms behind this beneficial effect of liraglutide are uncertain. However, the treatment effect of liraglutide commenced after 6–9 months of treatment, similar to what is seen in statin trials, which may indicate an effect on the atherosclerotic process. Several animal studies have been performed to elucidate the mechanisms underlying the beneficial cardiovascular effects of liraglutide, but so far, no human data are available.

Coronary computed tomography angiography (CCTA) is a non-invasive method that enables investigation of atherosclerotic coronary plaque composition and progression [4]. Substantial evidence indicates that plaque composition plays an important role in the development of acute coronary syndrome [5]. In the present observational study, we evaluated changes in coronary artery plaque volumes and composition using serial CCTA in cardiac asymptomatic patients with T2D receiving liraglutide (Lira+) compared to cardiac asymptomatic patients with T2D not receiving liraglutide (Lira-).

## Research design and methods

### Study design and population

This study was a single-center prospective observational study performed from March 2016 to September 2017 at Odense University Hospital Svendborg, Svendborg, Denmark. The data presented here were collected as part of a natural history study of coronary atherosclerosis in asymptomatic patients with T2D. Patients with T2D and no clinical indication for a CCTA were invited to participate and all examinations were performed at baseline and repeated at follow-up one year later. Patients were required to be cardiac asymptomatic and without a history of coronary artery disease.

### Criteria for participation

Inclusion criteria were age of at least 18 years, T2D, and written informed consent. Exclusion criteria were presence of angina, history of CVD, documented heart failure, estimated glomerular filtration rate (eGFR) < 45mL/min/1.73m^2^, allergy to iodine contrast, liraglutide treatment < 1 year, treatment with other GLP-1 receptor agonists, and non-interpretable CCTA.

### Data collection

Data on baseline characteristics such as age, gender, height, weight, body mass index (BMI), smoking status, smoking exposure in terms of pack years, and duration of diabetes were collected. Data from blood samples including glycated hemoglobin A1c (HbA1c), low-density lipoprotein (LDL) cholesterol, high-density lipoprotein (HDL) cholesterol, triglycerides, eGFR, and high-sensitivity C-reactive protein (hsCRP) were also collected. Furthermore, data on current medications, dosage, and duration were registered. Medications included insulin, metformin, dipeptidyl peptidase 4 inhibitors, sulfonylurea, sodium-glucose co-transporter-2, acetylsalicylic acid beta-adrenergic receptor antagonist (beta-blocker), angiotensin-converting enzyme (ACE) inhibitor, aldosterone receptor blocker (ARB), lipid-lowering agents (HMG-CoA-reductase inhibitors and ezetimibe). Medication status was based on data from the baseline visit. Duration of lipid-lowering medication and liraglutide were registered. Lipid profile and BMI measurements were repeated at follow-up. Patients receiving one or more antihypertensive medications were categorized as having hypertension, whereas patients treated with one or more lipid-lowering medications were categorized as having hypercholesterolemia. CCTA-derived data such as the coronary artery calcium (CAC), total atheroma volume (TAV), dense calcium, fibrous, fibrous fatty, and necrotic core plaque volume were acquired as described below.

### Coronary computed tomography angiography

Patients were prepared for the CCTA with tablet ivabradine 7.5 mg once daily two days before the scan. If needed, an intravenous beta-blocker was administered on the day of the scan in patients with heart rate > 65 beats/minute [6]. Initially, an unenhanced scan was performed to assess the CAC based on the Agatston score. Sublingual fast-acting nitrate was administered shortly before the enhanced scan. Images were obtained by a 256-detector system (GE revolution CT, Waukesha, Wisconsin, USA) by an electrocardiogram-gated prospective acquisition in the 75% of the R-R interval with additional padding of 45 ms to allow additional reconstruction. An additional phase was acquired in the 40% phase of the R-R interval in patients with heart rates above 65 beats/minute. Furthermore, a repeated scan was acquired if the heart rhythm was irregular. A fixed volume of 60 mL of iodine contrast (Visipaque 320 mg iodine/mL) was injected at 5 mL/second, and the scan was performed when maximal attenuation was detected in the ascending aorta. The tube voltage and current were modulated according to patient BMI with a tube voltage between 80 and 140 kV and a tube current between 150 and 700 mA according to BMI. Gantry rotation time was 280 ms with 16 cm of axial coverage. The median radiation dose per scan was 2.01 millisievert. The slice thickness was 0.625 mm, and 40% adaptive statistical iterative reconstruction was adopted. All available phases were reconstructed and images with superior image quality were selected for analysis. Reconstructed images were analyzed on an offline workstation with validated semiautomatic software (Qangio CT Research Edition version 3.1.3.18, Medis, Leiden, NL) [7]. All images were analyzed by a single experienced observer blinded to patient data. Plaque location was designated according to the American Heart Association modified 17-segment model [8].

### Quantitative CTTA analysis

**Fig. 1:**
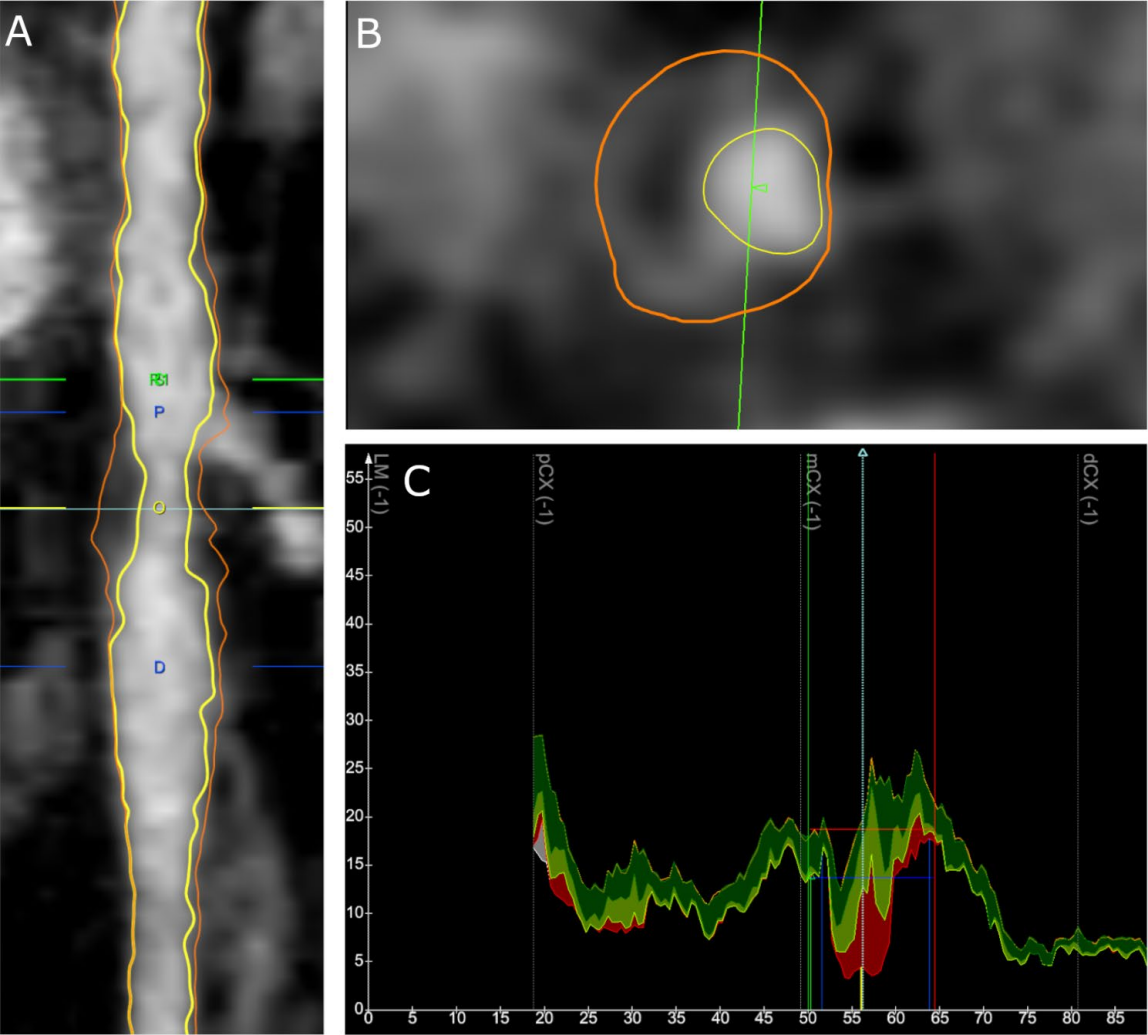
Quantitative plaque analysis in the left circumflex coronary artery Longitudinal straightened multiplanar reconstruction **(A)**. Transverse vessel view of a non-calcified plaque **(B)**. Graph depicting lumen and vessel area as a function of length. The total atheroma volume was segmented into plaque subtypes dense calcium (grey), fibrous (dark-green), fibrous-fatty (light-green), and necrotic core plaque (red) based on Hounsfield units **(C)**

Centerlines of each coronary artery were automatically extracted by the software. Based on longitudinal images, cross-sectional lumen and vessel contours were created and manually corrected by the observer, if necessary. Segments with insufficient image quality were excluded from analysis, and the software automatically excluded segments with a lumen diameter less than 1.5 mm. All available segments were screened for the presence of plaque, and segments without visible plaque were excluded from the analysis. For each TAV (total vessel volume - total lumen volume) was calculated (Fig. 1). Furthermore, the volumes of plaque types including dense calcium, fibrous, fibrous–fatty, and necrotic core plaque were calculated based on Hounsfield units. As plaque attenuation values are highly influenced by luminal contrast densities, the software applied a dynamic algorithm that adapted the Hounsfield unit thresholds according to luminal contrast densities [9]. Changes in plaque parameters measured from baseline to follow-up CCTA were calculated to assess the influence of liraglutide on longitudinal plaque changes. To address the differences in length of vessels analyzed in Lira + and Lira- patients, TAV and volumes of fibrous, fibrous-fatty, necrotic core, and necrotic core plaque were normalized according to vessel length by the following formula: (plaque volume/segment length) × mean segment length population.

### Study endpoints

The primary endpoint was change in TAV from baseline to follow-up stratified by liraglutide treatment. Secondary endpoints were to investigate changes in each plaque sub-type from baseline to follow-up stratified by liraglutide treatment.

### Statistical analysis

STATA IC 14.2 was used for statistical analysis. Baseline parameters are presented as means with corresponding standard deviation, counts with corresponding percentages, and medians with inter-quartile range (IQR). Student’s *t* test was performed to assess the differences of means between the two groups (Lira + and Lira-). A chi-square test was performed to assess the difference in proportions between the two groups (Lira + and Lira-). A two-sided *P* value of < 0.05 was considered statistically significant. Data on dense calcium volume, changes in dense calcium from baseline to follow-up, and the CAC score followed a non-normal distribution, and the Kristal-Wallis one-way analysis of variance was used to test if medians between groups were equal. A multivariate linear regression model was used to assess the association of changes in TAV and liraglutide treatment with adjustment of BMI, duration of diabetes, proportion of insulin users, and duration of lipid-lowering treatment, triglyceride levels, and Hb1Ac. A univariate linear regression model was used to assess the changes in TAV with adjustment of liraglutide treatment duration in years. For the linear regression models, assumptions including linearity, homoscedasticity of residuals, and normality were met. Results of the linear regression models were reported as regression coefficient (β) and 95% confidence interval (CI). Inter- and intra-reader agreement were assessed by Pearson’s correlation coefficient (Pearson’s *r*) and bland Bland-Altman analysis using 95% limits of agreement (95% LOA) [10] for twenty random vessels.

## Results

### Patient characteristics

Two hundred and sixty-one patients with T2D were eligible for participation. A total of 209 patients had diagnostic CCTA at baseline and follow-up of whom further 5 patients were excluded due to liraglutide treatment less than one year. The causes for exclusion were non-diagnostic CCTA baseline (n = 25), baseline CCTA not performed (n = 5), diagnosed with type 1 diabetes (n = 1), lost to follow-up (n = 15), contrast reaction (n = 4), non-cardiac death (n = 1) and non-diagnostic CCTA at follow-up (n = 1). A total of 204 patients were included in the final analysis, of whom 55 (27%) were treated with liraglutide, and 149 (73%) patients did not receive treatment with liraglutide. The mean CCTA scan interval was 13 months. The mean duration of liraglutide treatment was 4.6 ± 1.9 years and ranged from 1.0 to 7.4 years. The median and mean dose of liraglutide in the Lira + group was 1.2 mg IQR (1.2–1.2) and 1.3 ± 0.35 mg. respectively. The mean age was 61 ± 10 years and 149 (73%) of the study patients were men (Table 1). The proportion of patients with dyslipidemia (78% (Lira+) vs. 79% (Lira-), *P* = 0.88), and hypertension (66% (Lira+) vs. 75% (Lira-), *P* = 0.21), was equal in the two groups. There was no difference in the proportion of active smokers (18% (Lira+) vs. 25% (Lira-), *P* = 0.32) or the exposure of smoking in terms of pack years (14.5 ± 16.5 (Lira+) vs. 16.4 ± 20.0 years (Lira-), *P* = 0.52). In the Lira + group more patients were treated with insulin (51% (Lira+) vs. 35% (Lira-), *P* = 0.04) and metformin (98% (Lira+) vs. 80% (Lira-), *P* = 0.02), and the patients in the Lira + group had significantly longer duration of lipid-lowering treatment (8.1 ± 5.6 (Lira+) vs. 5.8 ± 5.8 years (Lira-), *P* = 0.01). Furthermore, patients in the Lira + group had significantly higher BMI (32.1 ± 4.8 (Lira+) vs. 29.9 ± 4.8 kg/m^2^ (Lira-), *P* = 0.002). Diabetes duration was longer in the Lira + group (13.5 ± 7.0 (Lira+) vs. 9.7 ± 8.9 years (Lira-), *P* = 0.003), and the Lira + group had significantly higher triglycerides (2.3 ± 1.2 (Lira+) vs. 1.9 ± 0.9 mmol/L (Lira-), *P* = 0.02). Furthermore, the Lira + group had significantly higher levels of HbA1c (65 ± 16 (Lira+) vs. 58 ± 13 mmol/mol (Lira-), *P* = 0.003).

**Table 1:** Baseline demographics of 204 patients stratified by liraglutide treatment

	All patients (n = 204)	Lira+ (n = 55)	Lira- (n = 149)	*P* value
Age (years)	61 ± 10	61 ± 9	61 ± 10	0.844
Male, n (%)	149 (73)	38 (69)	111 (74)	0.440
Body mass index (kg/m^2^)	30.5 ± 4.6	32.1 ± 4.8	29.9 ± 4.8	**0.002**
Hypertension, n (%)	139 (68)	41 (75)	98 (66)	0.214
Dyslipidemia, n (%)	161 (79)	43 (78)	118 (79)	0.875
Active smokers, n (%)	47 (23)	10 (18)	37 (25)	0.317
Pack years (years/20 cigarettes daily)	15.9 ± 19.1	14.5 ± 16.7	16.4 ± 20.0	0.519
Duration of diabetes (years)	10.8 ± 7.0	13.5 ± 7.0	9.7 ± 8.9	**0.003**
Medication at baseline				
- Insulin	80 (39)	28 (51)	52 (35)	**0.038**
- Biguanide	169 (82)	51 (98)	118 (80)	**0.023**
- DPP-4 inhibitor	29 (14)	1 (1)	28 (19)	**0.002**
- Sulfonylureas	36 (18)	13 (24)	23 (15)	0.173
- SGLT2 inhibitor	18 (9)	5 (9)	13 (9)	0.935
- Acetylsalicylic acid	24 (12)	5 (9)	19 (13)	0.471
- Beta-blocker	18 (9)	6 (11)	12 (8)	0.523
- ACE inhibitor/ ARB	131 (64)	38 (69)	93 (63)	0.779
- Lipid-lowering, n (%)	154 (75)	44 (80)	110 (74)	0.363
- Duration of lipid-lowering (years)	6.5 ± 5.8	8.1 ± 5.6	5.8 ± 5.8	**0.009**
Laboratory findings				
- LDL (mmol/L)	2.0 ± 0.8	1.9 ± 0.8	2.0 ± 0.8	0.482
- HDL (mmol/L)	1.2 ± 0.3	1.2 ± 0.3	1.2 ± 0.4	0.434
- Triglycerides (mmol/L)	2.0 ± 1.0	2.3 ± 1.2	1.9 ± 0.9	**0.022**
- HbA1c (mmol/mol)	60.2 ± 14.2	65.0 ± 15.7	58.4 ± 13.2	**0.003**
- hsCRP (mg/L)	2.8 ± 3.5	2.9 ± 3.6	2.7 ± 3.5	0.726
CAC score (Agatston units)	416 IQR (0-481)	509.1 IQR (0-481)	381 IQR (1-460)	0.297

Continuous variables are expressed as mean with corresponding standard deviation or counts with the corresponding percentage. CAC score is presented as the median and interquartile range (IQR).

Lira+, Liraglutide treated patients; Lira-, Patients not treated with Liraglutide; DPP-4, Dipeptidyl peptidase-4; SGLT2, sodium-glucose co-transporter 2; ACE, angiotensin-converting-enzyme; ARB, angiotensin-2 receptor blocker; LDL, low-density lipoprotein; HDL, high-density lipoprotein; HbA1c, glycated hemoglobin A1c; hsCRP, high-sensitive C-reactive protein; CAC, coronary artery calcium.

### CCTA results

**Fig. 2:**
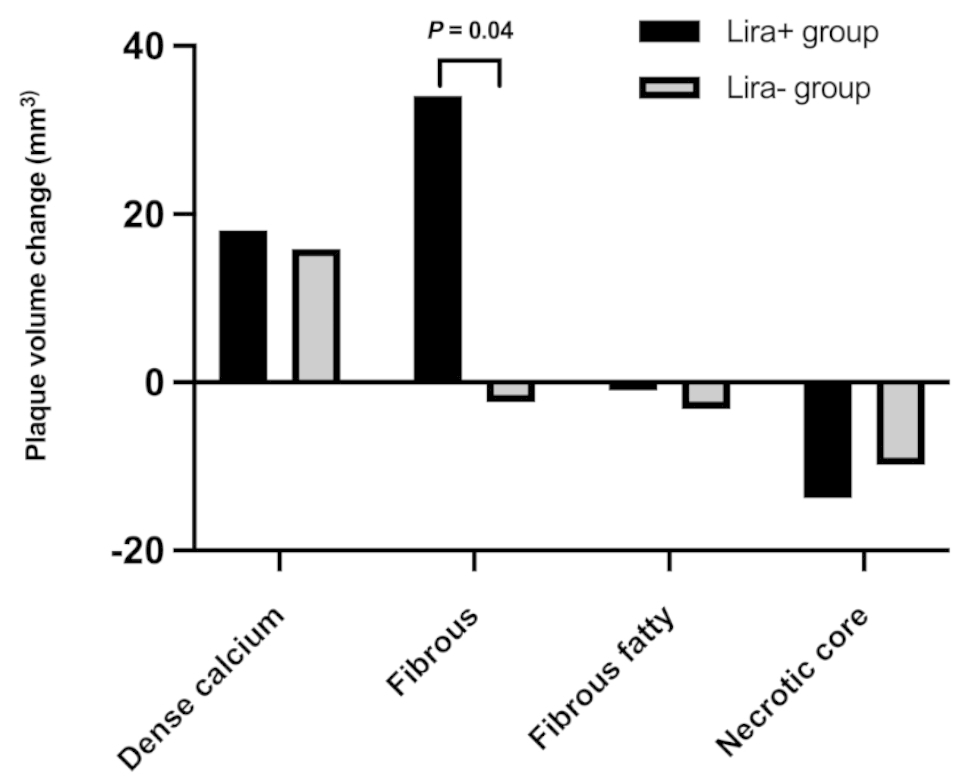
Normalized plaque volumes of dense calcium, fibrous, fibrous-fatty, and necrotic core plaque during 1-year follow-up in patients treated with liraglutide (Lira+) and patients not treated with liraglutide (Lira-)

Overall, TAV showed a considerable variability at baseline, but did not differ significantly between the two groups (599 ± 341 (Lira+) vs. 671 ± 359 mm^3^ (Lira-), *P* = 0.20) (Table 2). Baseline volumes of fibrous plaque were slightly higher in Lira- patients (347 ± 198 (Lira+) vs. 405.8 ± 211 mm^3^ (Lira-), *P* = 0.07) but the difference was not statistically significant. The other plaque subtypes were equal between groups at baseline. At follow-up, no significant changes in plaque volumes of TAV, dense calcium, fibrous-fatty, or necrotic core were detected. However, in the Lira + group, a significant increase in fibrous plaque (34 ± 129 (Lira+) vs. -2 ± 101 mm^3^ (Lira-), *P* = 0.04) was found (Fig. 2).

**Table 2:** Changes in plaque stratified by liraglutide treatment

	Lira + (n = 55)	Lira - (n = 149)	*P* value
Total atheroma volume (mm^3^)			
- Baseline	599.4 ± 340.6	671.2 ± 358.8	0.200
- Follow-up	637.5 ± 386.6	669.9 ± 369.3	0.583
- Difference	38.0 ± 180.3	-1.3 ± 159.7	0.134
Dense calcium plaque volume (mm^3^)			
- Baseline	37.7 IQR (5.8–89.6)	40.0 IQR (4.5–114.3)	0.956
- Follow-up	41.9 IQR (5.0–139.9)	47.7 IQR (6.0–141.0)	1.000
- Difference	6.7 IQR (0–26.3)	7.3 IQR (0–23.5)	0.959
Fibrous plaque volume (mm^3^)			
- Baseline	346.5 ± 198.4	405.8 ± 211.2	0.072
- Follow-up	380.6 ± 248.4	403.4 ± 218.5	0.524
- Difference	34.1 ± 128.7	-2.4 ± 101.0	**0.035**
Fibrous fatty plaque volume (mm^3^)			
- Baseline	98.0 ± 62.7	113.2 ± 77.3	0.192
- Follow-up	97.0 ± 60.7	110.0 ± 79.5	0.272
- Difference	-1.0 ± 45.4	-3.2 ± 40.3	0.739
Necrotic core plaque volume (mm^3^)			
- Baseline	59.7 ± 72.2	58.9 ± 75.7	0.949
- Follow-up	45.4 ± 50.0	49.5 ± 65.1	0.675
- Difference	-13.8 ± 69.0	-9.8 ± 61.4	0.695

Continuous variables are expressed as mean with corresponding standard deviation. Non-normally distributed data are presented as the median and interquartile range (IQR). Lira+, liraglutide-treated group; Lira-, non-liraglutide-treated group.

### Linear regression models

In a multivariate regression model, treatment with liraglutide (adjusted for potential confounders including BMI, duration of diabetes, insulin use, duration of lipid-lowering treatment, triglyceride levels, and Hb1Ac) was not associated with a change in TAV (β 39.0, 95% CI -16.7 ; 94 − 7, *P* = 0.17). The regression coefficients for each confounder in the multivariate analysis are shown in Table 3. Duration of liraglutide treatment before the study was not associated with a change in TAV (β -3.6 95% CI: -54.0 ; 56.9, *P* = 0.89).

**Table 3:** Multivariate linear regression of change in total atheroma volume (n = 204)

Variable	Coefficient (β)	*P* value	95% CI for β
Liraglutide treatment	42.8	0.13	(-13.3–99.0)
Insulin use	7.0	0.80	(-46.7–60.7)
Diabetes duration, (years)	1.6	0.48	(-3.0–6.2)
Age, (years)	0.79	0.57	(-1.9–3.56)
Hb1Ac, (mmol/mol)	0.06	0.95	(-1.8–1.9)
Lipid-lowering treatment, (years)	-1.6	0.49	(-6.2–2.9)
Triglycerides, (mmol/L)	-2.5	-0.84	(-27.0–22.0)
BMI, (kg/m^2^)	-3.1	0.24	(-8.5–2.2)
Sex	-32.2	0.24	(-86.2–21.7)

BMI, body mass index; Hb1Ac, glycated hemoglobin A1c.

### Inter- and intra-observer variability

Repeatability was good, and the mean bias for intra-observer and inter-observer variability for TAV was 5.0 mm^3^, 95% LOA (-89.7 ; 99,7) and 8.5 mm^3^, 95% LOA (-176.1 ; 193.1). A strong correlation was detected between both intra-observer analysis (r = 0.98) and inter-observer analysis (r = 0.91). Inter- and intra-observer agreement and correlation for additional plaque components are shown in Table 4.

**Table 4:** Inter and intra-observer variability

Variable	Intra-observer variability Mean diff. [95% LOA]	r	Inter-observer variability Mean diff. [95% LOA]	r
Total atheroma volume	5.0 [-89.7 ; 99.7]	0.98	8.5 [-176.1 ; 193.1]	0.91
Dense plaque	1.6 [-22.9 ; 26.1]	0.99	0.5 [-28.9 ; 29.9]	0.99
Fibrous plaque	5.9 [-11.5 ; 23.2]	0.95	5.7 [-13.5 ; 24.9]	0.95
Fibrous-fatty plaque	1.1 [ -28.7 ; 30.9]	0.96	5.3 [-44.9 ; 55.4]	0.87
Necrotic core plaque	-3.9 [-45.7 ; 37.9]	0.91	-3.1 [-55.7 ; 49.4]	0.86

Mean diff, Mean difference between paired observations; LOA, Limits of agreement calculated as the mean difference ± 1.96 standard deviation; r, Pearson’s coefficient of correlation between paired observations.

## Discussion

In this prospective observational study, we assessed coronary atherosclerosis in asymptomatic patients with T2D treated with and without the GLP-1 receptor agonist liraglutide. TAV progression was similar in the two groups, but liraglutide-treated patients experienced a significantly higher rate of fibrous plaque progression.

The LEADER study showed that T2D patients with established or at high risk of CVD treated 1.8 mg with liraglutide once daily in addition to standard of care experienced significantly lower rates of major adverse cardiovascular events compared to placebo. In addition, patients receiving liraglutide achieved significantly better glycemic control and greater weight loss compared to the patients receiving placebo. However, no clear mechanistic explanation of the CVD-protective effect of liraglutide was exposed. In the LEADER study, the reduction in major adverse cardiovascular events commenced after 6–9 months of treatment, a phenomenon well known from statin treatment [11, 12]. Statins remain a cornerstone in CVD prevention due to their ability to halt plaque progression [13, 14] and modify plaque composition [15]. Thus, the reduction in cardiovascular mortality seen in the LEADER trial occurred beyond may be rooted in structural changes in the coronary artery plaque.

To the best of our knowledge, the present study is the first to assess the effect of liraglutide on coronary atherosclerosis in patients with T2D. Several studies have investigated the effect of GLP-1 and GLP-1 receptor agonists on the atherosclerotic plaque formation in animal studies. Burgmaier et al. demonstrated that GLP-1 metabolites GLP(7–37), GLP-1(9–37), and GLP-1(28–37) significantly reduced macrophage infiltration and matrix-metalloproteinase expression as well as increased collagen content and cap thickness in aortic plaque [16]. Other studies have demonstrated that liraglutide attenuates plaque progression and increases smooth muscles and collagen content in aortic plaque [17–19]. A single study examined the effect of exenatide on human coronary artery endothelial cells in vitro [20]. The authors showed that exenatide stimulated endothelial cell proliferation that may cover atherosclerotic lesions protecting from atherothrombotic events.

In the present study, both the Lira + group and the Lira- group experienced regression of necrotic core and fibrous fatty plaque volumes while only liraglutide-treated patients experienced progression of fibrous plaque volume.

Fibrous plaque serves an important role, and a thin fibrous cap lining the coronary artery lumen is a hallmark of ruptured plaques in autopsy studies [21]. The degradation of oxidized lipids in the plaque by macrophages forms foam cells that excrete matrix metalloproteinase leading to a loss of smooth muscle cells and collagen contributing to plaque instability [22]. Anholm et al. suggest that liraglutide is associated with reduced lipid oxidation leading to lower cardiovascular risk. In a randomized trial, the authors demonstrated that 12 weeks of liraglutide in combination with metformin treatment significantly reduced lipolysis compared to metformin and placebo [23]. Furthermore, the authors demonstrated that liraglutide in combination with metformin improved the atherogenic lipid profile and reduced CRP in patients with newly diagnosed T2D and stable coronary artery disease [24]. In the present study, CRP levels were equal between the Lira + and Lira- patients. Patients in the Lira + group had significantly higher glycated hemoglobin and BMI associated with low-grade inflammation and elevated CRP, and liraglutide treatment could have attenuated CRP in the Lira + group to the same level observed in the Lira- group [25].

The beneficial effects of statins are closely correlated with the reduction in LDL cholesterol, whereas the risk reduction seen in the LEADER trial is likely multifactorial and due to improvement of several risk factors including HbA1c, body weight, inflammation, and insulin resistance. Overall, the present study population was a low-risk population compared to the population in the Leader trial, and investigating the association of liraglutide and plaque composition in a high-risk population might provide different results. Another important limitation is that the Lira + group was characterized by a longer T2D duration, more insulin users, higher HbA1c and BMI compared to the Lira- group.

## Conclusions

Among several beneficial effects on the cardiovascular system, our findings may suggest that liraglutide prompts plaque stabilization by increased fibrous plaque possibly contributing to the improved CVD outcome in patients treated with liraglutide. The present study was a hypothesis-generating study, and further studies using appropriate study designs and statistical power are warranted to corroborate these findings.

## Data Availability

The data from the present study can be provided by the corresponding author on reasonable request.
